# Influence of different customized abutment materials on stress distribution of different internal implant-abutment connections: finite element analysis

**DOI:** 10.1186/s12903-025-07122-8

**Published:** 2025-11-22

**Authors:** Ahmed Ziada, Marwa Beleidy

**Affiliations:** 1 Department of Fixed Prosthodontics, Faculty of Dentistry, Benisuef University, Benisuef, Egypt; 2 Department of Fixed Prosthodontics, Faculty of Dentistry, October 6 University, Giza, Egypt

**Keywords:** Morse taper, Star-shaped tube-in-tube, CAD/CAM abutments, Implant-abutment connection, Principal stress, Principal strain, Von mises stress, And finite element analysis

## Abstract

**Background:**

Limited research evaluates how the type of implant-abutment connection and the material of the abutment together affect the biomechanical behavior of load transfer within both the implant components and the surrounding bone under axial and oblique loading conditions. This 3-dimensional (3D) finite element analysis (FEA) study aimed to provide biomechanical insights to assist clinicians in choosing optimal connection designs and abutment materials, enhancing implant longevity and clinical outcomes.

**Methods:**

Two internal implant-abutment connections were modeled in 3D: model (S), which is a star-shaped tube-in-tube design, and model (H), which is a Morse taper combined with an internal hex, both intended to support a mandibular first molar crown and its associated bone geometry. Four abutment materials (Titanium grade V/Ti, Co-Cr, soft-milled Co-Cr–Mo/Co-Cr-S, and zirconia/Zr) were examined using both connection designs. Each crown was subjected to two loading protocols: (1) 200 N vertically was applied at six occlusal points, and (2) 100 N obliquely (at 45º) was applied to three occlusal points on the buccal bevel of the buccal cusp. FEA was performed to analyze the maximum and minimum principal stresses and strains on the peri-implant bone, as well as the von Mises stresses on the implants, abutments, screws, and crowns.

**Results:**

Principal stresses and strains were predominantly concentrated in the crestal cortical bone. Under axial loading, stress values were similar across connection types. The highest stress was observed in the H (Zr) model (15.683 MPa) and the lowest in S (Ti) (14.265 MPa). Oblique loading caused higher compressive stresses, peaking at 99.06 MPa in the H (Co-Cr) model. In cancellous bone, stresses were lower, ranging from 0.12888 MPa for H (Ti) to 0.21535 MPa for S (Zr). The highest cortical strain was observed in S (Co-Cr) under oblique loading conditions, measuring 6700 με. Conversely, all models exhibited reduced cancellous elastic strain values, with the maximum strain recorded at 1200.0 με in the S (Co-Cr) axially and 980.0 με in the S (Co-Cr) obliquely. The von Mises stress was localized at the implant and abutment necks, with peak implant stress attaining 135.0 MPa in the S (Co-Cr) model under oblique loading. Titanium abutments demonstrated the lowest stress values consistently across various loading conditions. All models exhibited minimal directional screw deformation (3.897 µm axial; 1.257 µm oblique), demonstrating mechanical stability.

**Conclusions:**

Star-shaped tube-in-tube and hybrid Morse taper with internal hex connections showed similar stress patterns, with values below the titanium alloy's yield strength and safe bone stress levels. Oblique loading, however, produced cortical strains above the safe limit. Zirconia, Co-Cr, and soft-milled Co-Cr–Mo abutments had moderate stress distribution, while titanium showed the most favorable profile. Both connections caused minimal screw deformation, suggesting low loosening risk.

**Supplementary Information:**

The online version contains supplementary material available at 10.1186/s12903-025-07122-8.

## Background

The implant-abutment connection significantly influences the long-term clinical efficacy of dental implants [1]. These interfaces are typically categorized based on the geometric configuration of the coronal portion of the implant body [2]. Internal connections offer superior biological sealing and mechanical stability compared to external connections [3]. Numerous internal connection geometries have been developed, such as internal hexagonal, internal octagonal, conical, and tube-in-tube designs. These geometries yield a cold-welded interface with high friction levels [4, 5]. Under compressive loading, abutment settling reduces the microgap, allowing the two components to function as a single unit. This result limits microleakage while improving resistance to rotation and bending torque [6].

While readily available and clinically dependable, stock abutments exhibit restricted customization capabilities and may lead to inadequate emergence profiles and difficulties in cement removal, especially with subgingival margins [7, 8]. Customized abutments provide individualized emergence profiles and enhance adaptation to peri-implant mucosa, promoting esthetics and peri-implant tissue health [9]. Long-term randomized clinical trials have demonstrated that customized abutments yield comparably advantageous survival, biological stability, and aesthetic results, with follow-up durations reaching 13 years [10]. Furthermore, research comparing prefabricated abutments with those fabricated using computer-aided design and manufacturing (CAD/CAM) supports comparable survival rates and aesthetic results during short- and mid-term follow-up [11]. Nevertheless, other studies report no significant differences in plaque accumulation, probing depth, or aesthetic indices between customized and stock abutments after 1–3 years, indicating that material properties and connection design may have a more substantial biomechanical impact than customization alone [12].

Abutment material selection is critical in implant longevity, influencing mechanical stability, biological response, and clinical performance. Titanium has long been considered the gold standard due to its excellent mechanical strength, corrosion resistance, biocompatibility, and favorable osseointegration [13]. Despite these benefits, its metallic hue may detract from the anterior parts' aesthetics, and its higher elastic modulus than bone can occasionally help with stress shielding [14]. In contrast, cobalt-chromium (Co-Cr) abutments are more cost-effective and rigid than noble alloys, especially in light of the increased cost of gold. Although remarkable for their resistance to corrosion and wear, their grayish metallic color restricts their use in aesthetic applications, and biocompatibility concerns, such as tissue irritation and hypersensitivity, persist as challenges [15].

Soft-milled Cobalt-Chromium-Molybdenum (Co-Cr–Mo) alloys were developed to address these limitations. Integrating Co-Cr’s mechanical advantages with the precision of digital manufacturing results in superior surface finish, improved fit, and minimized marginal discrepancies, which may decrease the possibility of biological complications like peri-implantitis [16]. However, their long-term clinical performance is still inadequately studied compared to titanium and zirconia.

Zirconia abutments enhance esthetics in anterior regions due to their tooth-like color and translucency. Their biocompatibility is excellent, presenting a minimal risk of irritation or allergic reactions [17]. Nonetheless, their comparatively reduced fracture resistance when subjected to high occlusal forces limits their application in posterior areas or among patients exhibiting parafunctional habits [18].

Abutment material and connection design significantly impact how implant systems transfer mechanical loads among their constituent parts and the surrounding bone. Due to the increasing structural complexity brought about by advancements in implant-abutment connection design, it is now more challenging to measure occlusal load transfer and peri-implant stress distribution. Insufficient studies have been conducted regarding the relationship between connection design and abutment material in implant biomechanics. To overcome these difficulties, finite element analysis (FEA) has been used extensively to simulate mechanical performance under regulated loading circumstances.

This study examined two under-researched connection configurations: the star-shaped tube-in-tube, the hybrid Morse taper with internal hex, and four customized abutment materials. Unlike previous studies that mainly focused on connection type and abutment material in isolation, a multifactorial FEA was performed to evaluate the impact of these variables on stress distribution within the implant system and surrounding bone. To improve implant longevity and clinical results, this study aimed to provide biomechanical insights to help clinicians choose optimum connection designs and abutment materials. According to the study, implant biomechanics would significantly impact the interplay between abutment material and connection design. The first hypothesis was that the hybrid Morse taper with internal hex would demonstrate lower crestal bone stress and strain. The second hypothesis was that the hybrid Morse taper with internal hex would reduce stress within implant components, compared with the star-shaped tube-in-tube connection, particularly under oblique loading. The third research hypothesis is that abutment material would modulate stress distribution within implant components, with soft-milled Co-Cr–Mo exhibiting a more favorable pattern than titanium, Co-Cr, and zirconia.

## Methods

The protocol for this study was approved by the Faculty of Dentistry, Beni-Suef University Research Ethics Committee (FDBSU-REC) (No. REC-FDBSU/0212025–04/ZA).

### Implant system designing and modeling

**Fig. 1:**
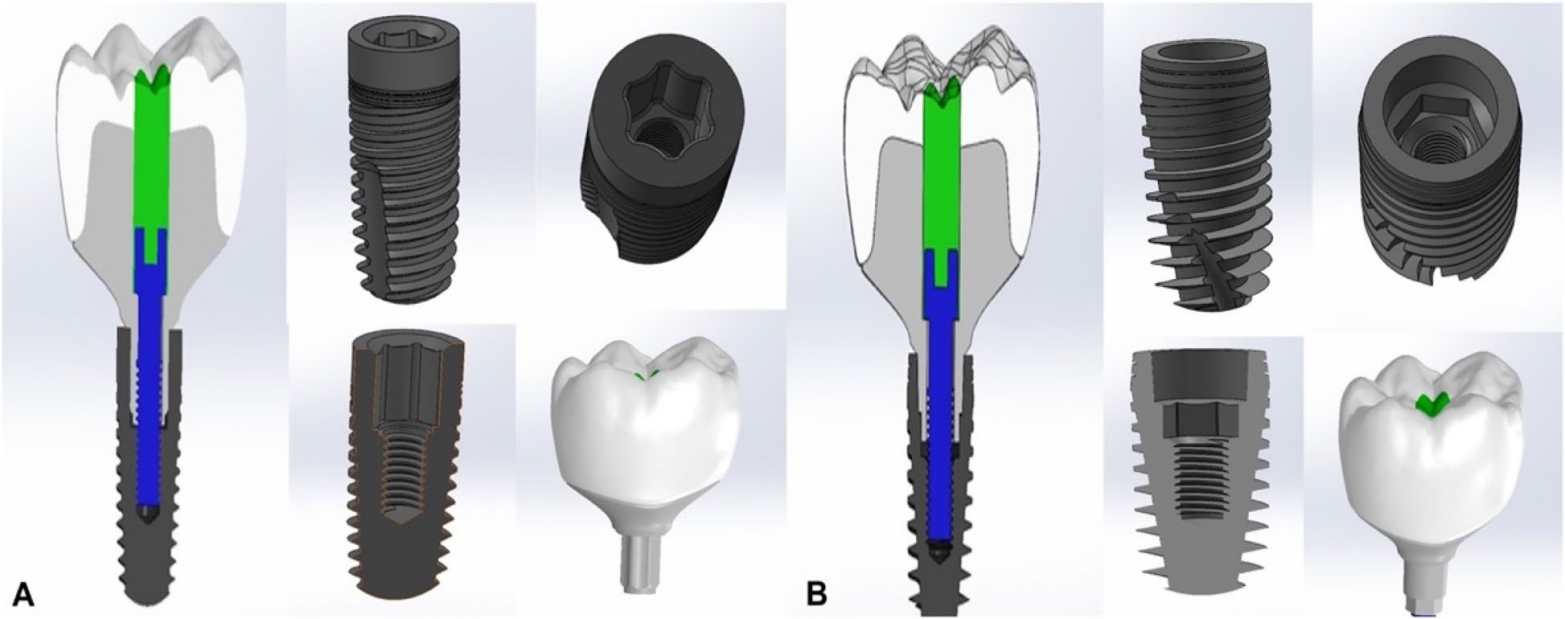
Finite element models of two tested implant systems: **A** Star-shaped tube-in-tube implant and **B** Hybrid 5° Morse taper combined with internal hex anti-rotation feature implant, showing the internal configuration and integrated assembly

Sixteen three-dimensional (3D) finite element models were established, consisting of an implant complex and alveolar bone in the mandibular first molar region (Fig. 1 and Table 1). A segment of the mandible, extending from the second premolar to the second molar, was chosen to reflect the clinical condition accurately and enhance computational efficiency for the simulation. Two parameters were considered: the implant-abutment connection design, which includes the Star-shaped tube-in-tube connection (S) (Torx®, Tube-in-Tube, Classic Sky, Bredent GmbH, Germany) and the Hybrid 5° Morse taper combined with an internal hex anti-rotation feature (H) (Conexa, Ev Line, B&B Dental S.r.l, Italy), as well as the abutment materials: Titanium Grade V (CORiTEC Titan Grade 5; pritidenta® GmbH, Leinfelden-Echterdingen, Germany), Co-Cr (Remanium Star MD II, Dentaurum, Germany), soft-milled Co-Cr–Mo (Ceramill Sintron, Ammann Girrbach, Austria), and Zirconia (Ceramill Zolid HT +, Ammann Girrbach, Austria). The exact diameter and length (4.1 mm × 10 mm) were used for each implant system. Both implants were digitally reconstructed using a reverse engineering technique to ensure precise replication of the implant geometry, thread design, platform connection, and screw. The abutments and crowns were designed using dental CAD software (exocad Dental CAD v3.0; exocad GmbH, Darmstadt, Germany) to ensure precise adaptation and optimal biomechanical performance. The thickness of the cement layer was set at 50 µm.

**Table 1:** Finite element models

Model	Connection type	Abutment material	Loading type
S1	Star-shaped tube-in-tube (S)	Titanium (Ti)	Axial
S2	Star-shaped tube-in-tube (S)	Co-Cr (Co-Cr)	Axial
S3	Star-shaped tube-in-tube (S)	Soft-milled Co-Cr–Mo (Co-CrS)	Axial
S4	Star-shaped tube-in-tube (S)	Zirconia (Zr)	Axial
S5	Star-shaped tube-in-tube (S)	Titanium (Ti)	Oblique
S6	Star-shaped tube-in-tube (S)	Co-Cr (Co-Cr)	Oblique
S7	Star-shaped tube-in-tube (S)	Soft-milled Co-Cr–Mo (Co-CrS)	Oblique
S8	Star-shaped tube-in-tube (S)	Zirconia (Zr)	Oblique
H9	Hybrid Morse taper with internal hex (H)	Titanium (Ti)	Axial
H10	Hybrid Morse taper with internal hex (H)	Co-Cr (Co-Cr)	Axial
H11	Hybrid Morse taper with internal hex (H)	Soft-milled Co-Cr–Mo (Co-CrS)	Axial
H12	Hybrid Morse taper with internal hex (H)	Zirconia (Zr)	Axial
H13	Hybrid Morse taper with internal hex (H)	Titanium (Ti)	Oblique
H14	Hybrid Morse taper with internal hex (H)	Co-Cr (Co-Cr)	Oblique
H15	Hybrid Morse taper with internal hex (H)	Soft-milled Co-Cr–Mo (Co-CrS)	Oblique
H16	Hybrid Morse taper with internal hex (H)	Zirconia (Zr)	Oblique

### Mandibular bone block model

**Fig. 2:**
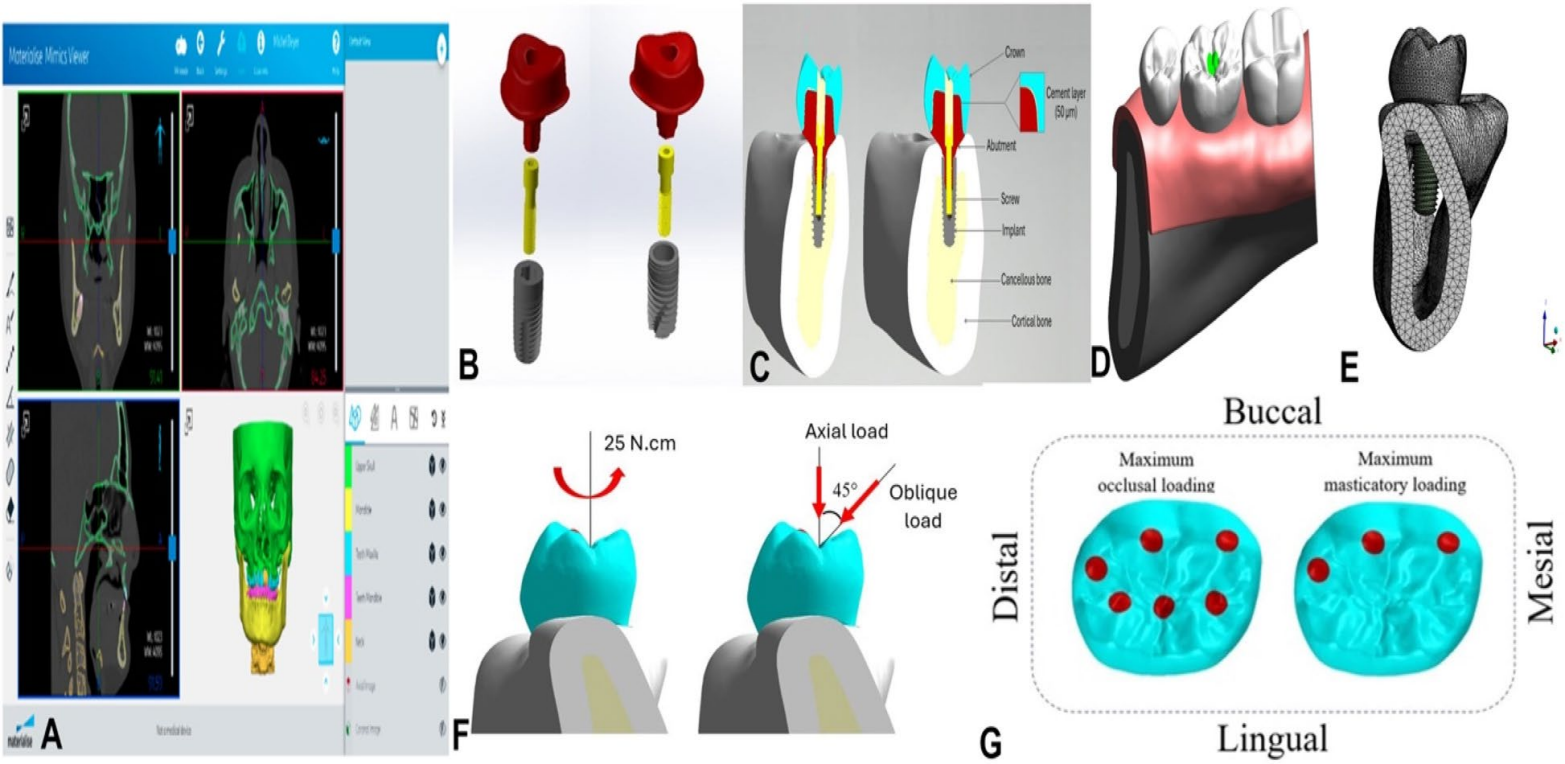
Finite element model construction: **A** 3D mandible creation and segmentation with thresholding from CBCT data; **B** Unassembled models; **C** Assembled models. **D** 3D assembly; **E** 3D CAD model meshing; **F** Loading orientations; and **G** Loading locations

The mandibular bone block model was reconstructed based on the cross-sectional images of the right first molar region obtained from cone beam computed tomography (CBCT) images (Mimics 21.0; Materialise, Leuven, Belgium) (Fig. 2A). The bone tissue comprised cancellous bone in the center, surrounded by 2 mm of cortical bone. The segmented 3D model (Fig. 2B) was exported in Standard Tessellation Language (STL) format and further refined (Geomagic Design X 2024.3.1; 3D Systems, SC, USA) to ensure smooth surfaces and correct any geometric errors before final processing. The segmented bone, implant, abutment, and crown components were assembled in a 3D model (SolidWorks 3D CAD; SolidWorks Dassault Systèmes Corp., MA, USA) (Fig. 2C and D) to ensure precise anatomical representation. The Boolean operation functions facilitated the integration of the mandibular bone block model with the dental implant model (Magics 20.03; Materialise, Leuven, Belgium).

### Material properties of assembly models

The material properties of the model components were defined according to data from the literature and summarized in Table 2 ([19–24], https://www.dentaurum.de/files/989-300-23.pdf, https://store.amanngirrbach.com/produkt/cad-cam-materialien/metall/ceramill-sintron/ceramill-sintron-98x12-f/?no_redirect=1, [25]). All materials were assumed to be homogeneous, linearly elastic, and isotropic.

**Table 2:** Material properties of the finite element models

Material	Modulus of elasticity (MPa)	Poisson's ratio	Reference
Zirconia crown	20000	0.31	[19]
Cement	7700	0.24	[20]
Cortical bone	13700	0.30	[21]
Cancellous bone	1370	0.30	[21]
Gingiva	3.0	0.45	[22]
Titanium abutment (Grade V)	114000	0.33	[23]
Zirconia abutment	206300	0.25	[24]
Co-Cr abutment	202000	0.31	(https://www.dentaurum.de/files/989-300-23.pdf)
Soft-milled Co-Cr abutment	200000	0.31	(https://store.amanngirrbach.com/produkt/cad-cam-materialien/metall/ceramill-sintron/ceramill-sintron-98x12-f/?no_redirect=1)
Titanium implant (Grade IV)	107000	0.33	[25]
Titanium abutment screw (Grade IV)	107000	0.33	[25]

### Interface conditions

A "frictional" contact condition was applied at the bone-implant connection to replicate the integration between the implant and the peri-implant bone in immediate placement scenarios. The interface between the prosthesis and the gingiva was modeled as bonded to facilitate realistic transmission of masticatory forces. Surface contacts were defined at the implant-abutment, abutment-screw, and abutment-cement interfaces, with frictional behavior determined by the quality of surface finishing. The assigned friction coefficients for these interfaces were 0.16, 0.441, and 0.25, respectively [26]. All other contact surfaces in the model were considered fully bonded [27].

### Meshing

The assembled models were imported into engineering simulation software (ANSYS Mechanical APDL Element Referenc; Release 18.2; ANSYS, PA, USA) to generate 10-node tetrahedral elements (Solid187) for the following simulation and calculations. A refined parabolic tetrahedral mesh with an element size of approximately 0.2 mm was applied in the regions surrounding the implant and peri-implant bone. In contrast, a coarser mesh was implemented for the adjacent soft tissues to maintain geometric accuracy and computational efficiency (Fig. 2E). Each model's total node and element numbers were recorded to ensure consistent analysis conditions (Table 3). Mesh convergence was evaluated through iterative refinement until the relative variation in maximum von Mises stress fell below 1%, signifying sufficient mesh resolution. It ensured both numerical stability and model reliability [28, 29].

**Table 3:** The total number of elements and nodes for each implant system

Model	Nodes	Elements
Star shaped tube-in-tube implant system (S)	415002	264239
Hybrid Morse taper with internal hex anti-rotation feature implant system (H)	412692	262563

### Loads and boundary constraints

All models were subjected to uniform loading and boundary conditions (Fig. 2F and G). The mesial, distal, cortical, and cancellous bone surfaces were constrained in the x, y, and z directions to eliminate displacement.

The simulation proceeded in two sequential steps (Fig. 2F). First, a preload corresponding to a screw tightening torque of 25 N.cm was applied, calculated using Bickford's formula, to simulate initial fixation. Next, functional masticatory forces were applied to the crown. A load of 200 N was distributed axially over 60 nodes encompassing three cusps and three fossae. To mimic non-axial loading, a 100 N force was used at a 45° buccolingual angle across 30 nodes on three buccal cusps (Fig. 2G). During load application, a reference point was established at the center of the abutment plane, with coupling constraints defined between this point and the coupling nodes on the abutment plane, ensuring that forces applied at the reference point were equivalently transmitted to the coupling nodes.

### Finite Element Analysis (FEA)

FEA was conducted (ANSYS Mechanical APDL Element Reference; Release 18.2; ANSYS, PA, USA). The maximum and minimum principal stresses and strains in the peri-implant bone were assessed. Positive values represented tensile stresses, whereas negative values denoted compressive stresses. The peaks of von Mises stress were examined to evaluate the biomechanical reactions of the implant components, including the implant body, abutment, implant-abutment connection, screw, and crown. Deformation analysis was performed along the Z-axis to simulate screw loosening, evaluating displacement under various loading conditions across all models.

## Results

### Bone stress and strain distribution

**Fig. 3:**
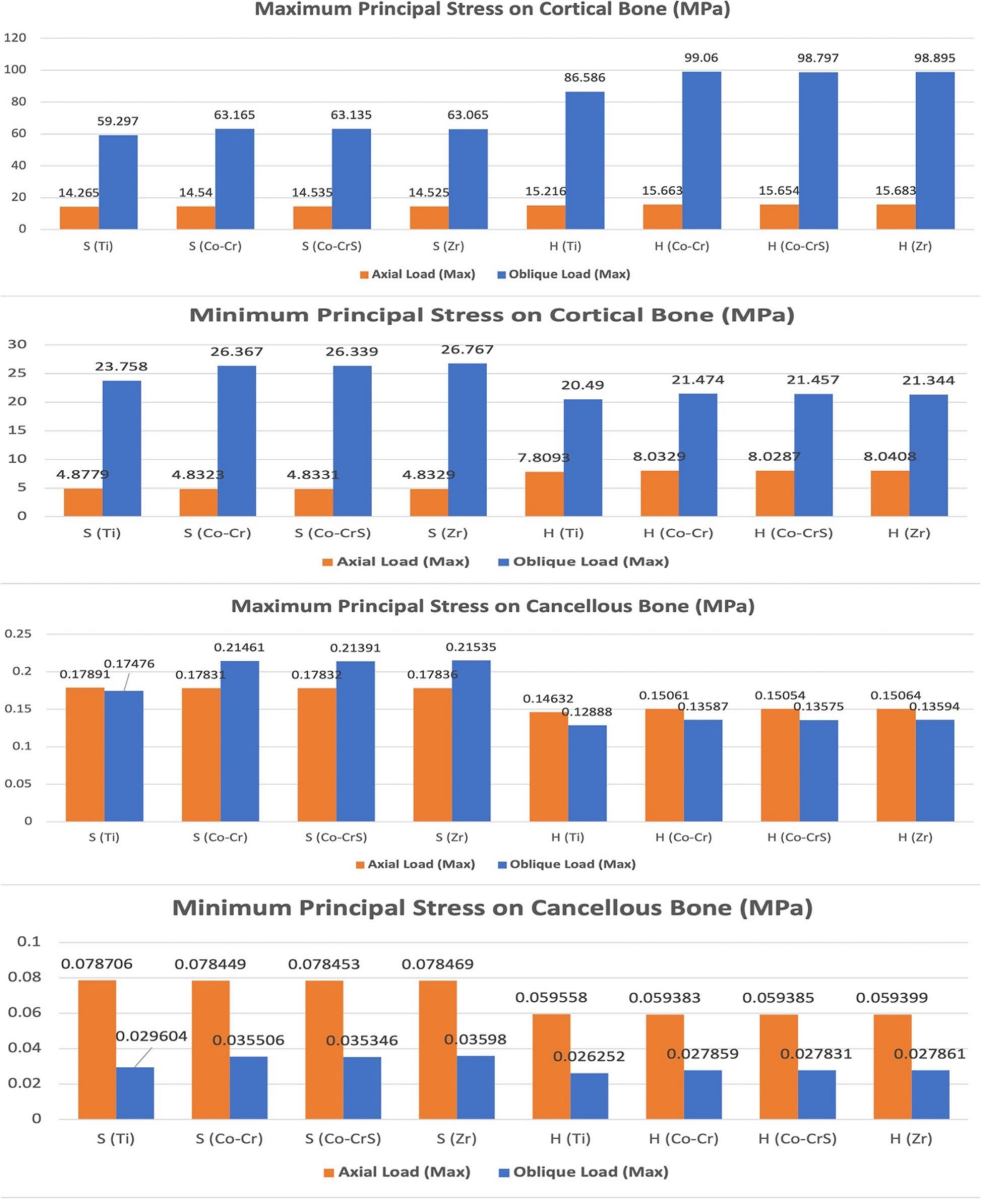
Chart illustrating the variation in maximum and minimum principal stress (MPa) recorded on the peri-implant bone (cortical and cancellous) of the 16 models under axial and oblique loadings

Maximum and minimum principal stresses were predominantly concentrated in the cortical crestal bone (Fig. 3). Under axial loading, both implant-abutment connections exhibited similar cortical bone stress magnitudes, with peak values ranging from 14.265 MPa S (Ti) to 15.683 MPa H (Zr). Oblique loading caused a significant increase in cortical bone stress, especially in the hybrid Morse taper with internal hex models, which reached peak compressive stresses up to 99.06 MPa in H (Co-Cr) compared to 59.297 MPa in S (Ti).

Cancellous bone stresses were considerably lower than cortical bone stresses across all models and loading conditions (Fig. 3). In axial loading, stresses ranged from 0.14632 MPa for H (Ti) to 0.17891 MPa for S (Ti). Meanwhile, oblique loading stresses ranged from 0.12888 MPa for H (Ti) to 0.21535 MPa for S (Zr).

**Fig. 4:**
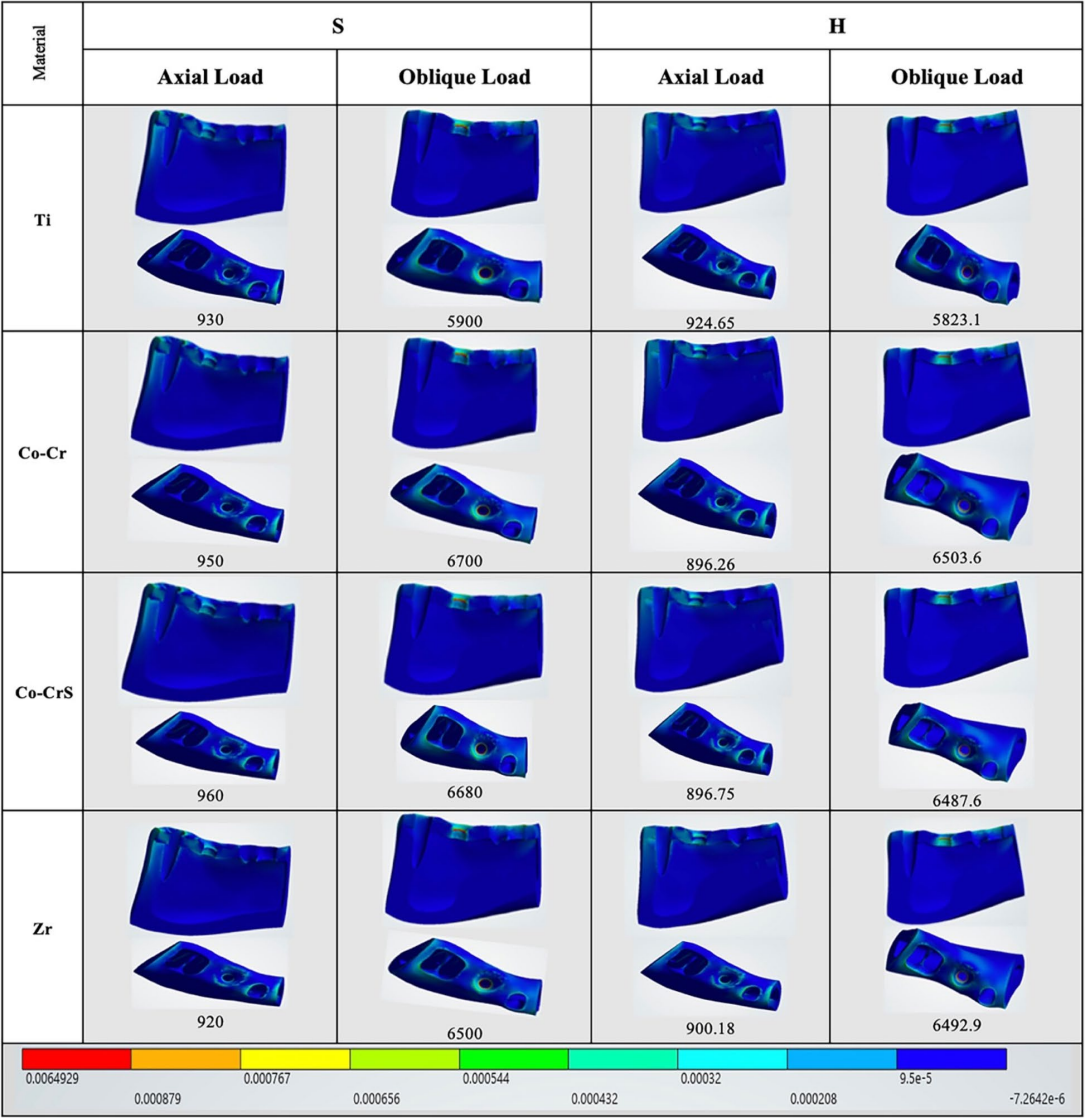
The distribution of the maximum principal strains on the cortical bone in the models with different connection designs and abutment materials for two loading conditions

**Fig. 5:**
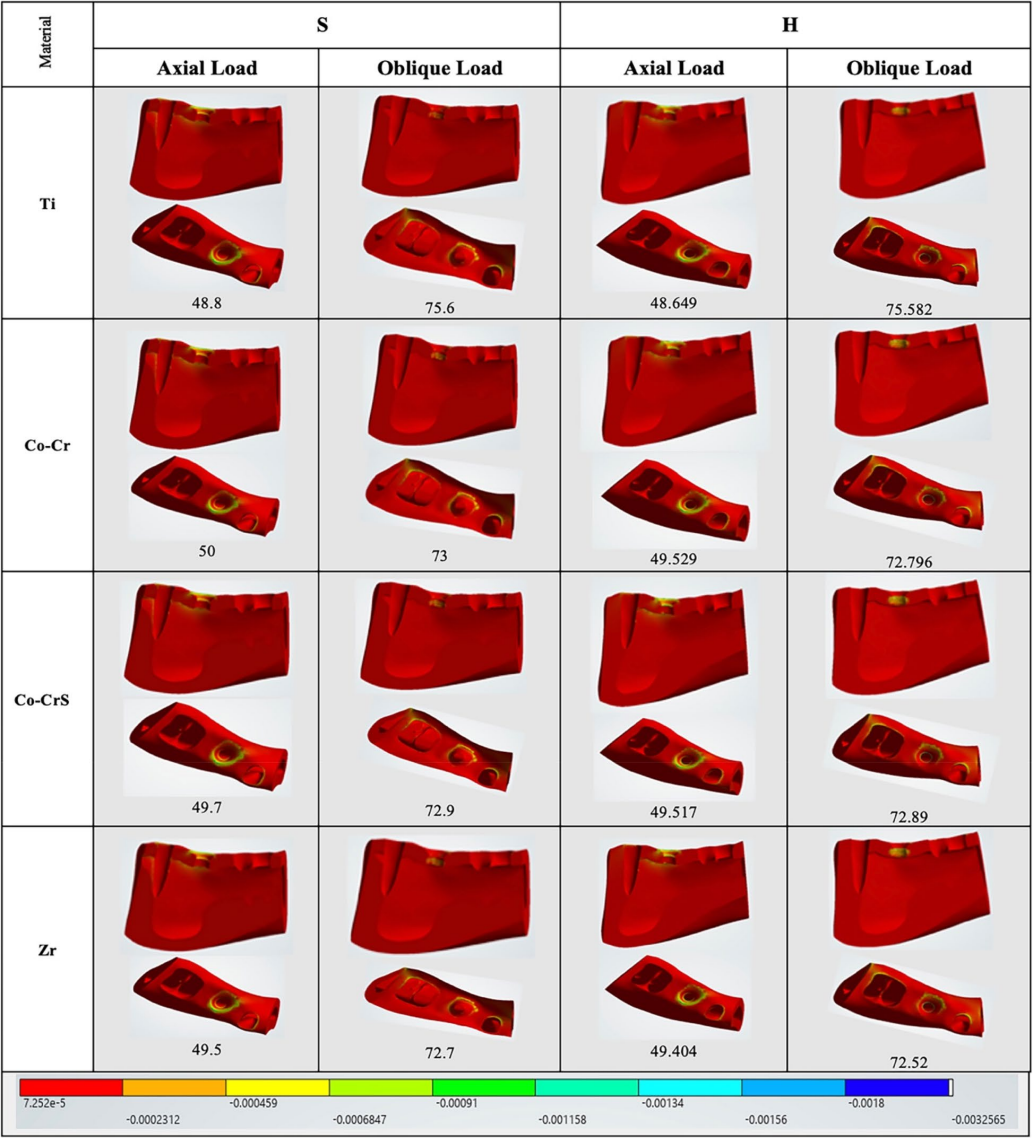
The distribution of the minimum principal strains on the cortical bone in the models with different connection designs and abutment materials for two loading conditions

**Fig. 6:**
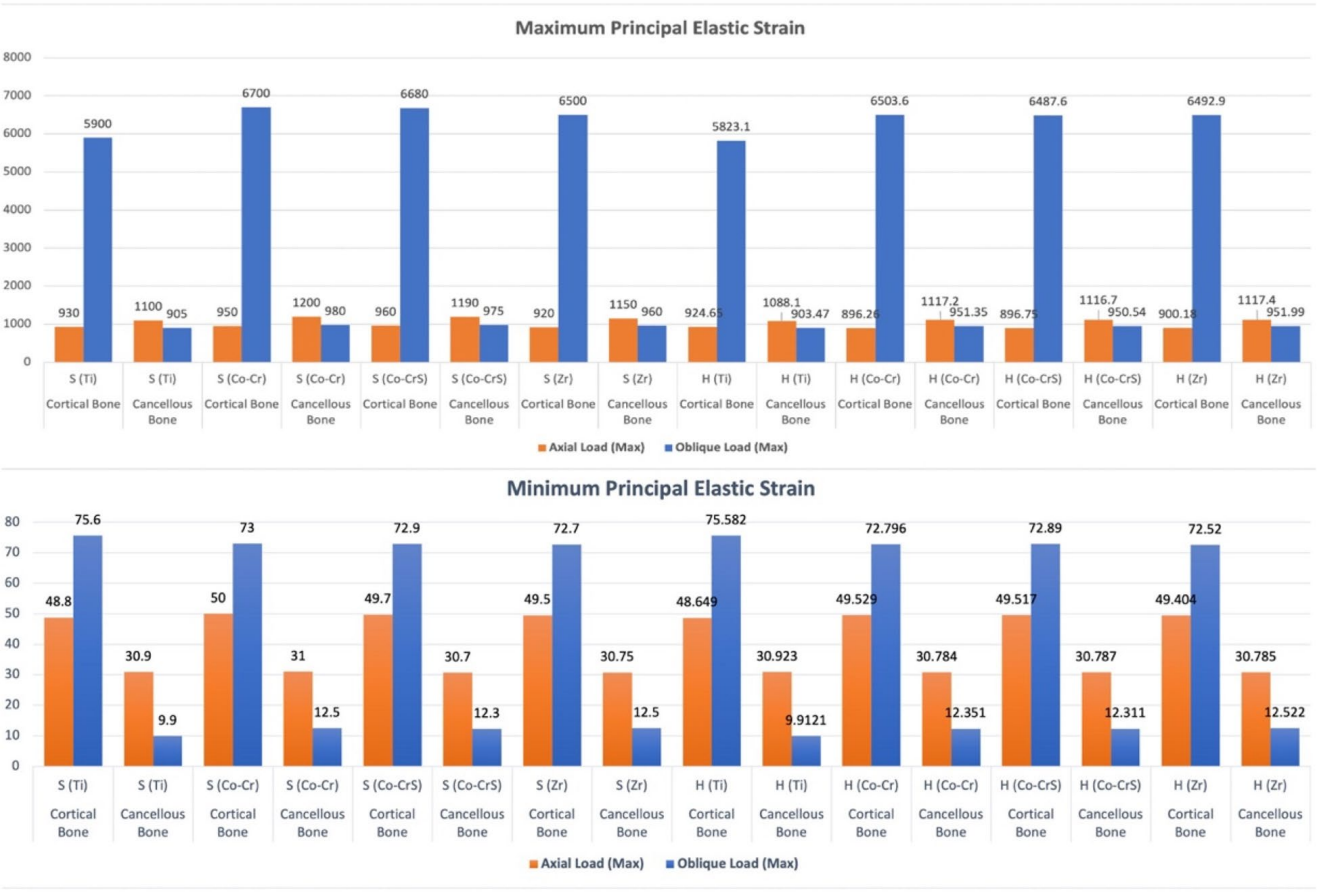
Chart illustrating the variation in maximum and minimum elastic strain (με) recorded on the peri-implant bone (cortical and cancellous) of the 16 models under axial and oblique loadings

Axial loading produced the highest elastic strain values in cortical bone in the S (Co-CrS) model (960.0 με). In comparison, the H (Co-Cr) and H (Co-CrS) models exhibited the lowest elastic strain values (896.26 and 896.75 με, respectively). Oblique loading significantly increased strain, peaking at 6700.0 με in the S (Co-CrS) model (Figs. 4, 5 and 6).

In cancellous bone (Fig. 6), strain values were generally lower and exhibited less variability, with the maximum axial strain (1200.0 με) recorded in the S (Co-Cr) model. Conversely, the H (Ti) exhibited the lowest values at 1088.1 με. Under oblique loading, all models showed lower elastic strain values. The highest strain (980.0 με) was observed in the S (Co-Cr), while H (Ti) had the lowest value (903.47 με).

The maximum and minimum stresses of cortical and cancellous bone and the strain distribution in cancellous bone are presented in supplementary figures (S1-S6) to complement the main text results.

### Implant, abutment, and crown stress

**Fig. 7:**
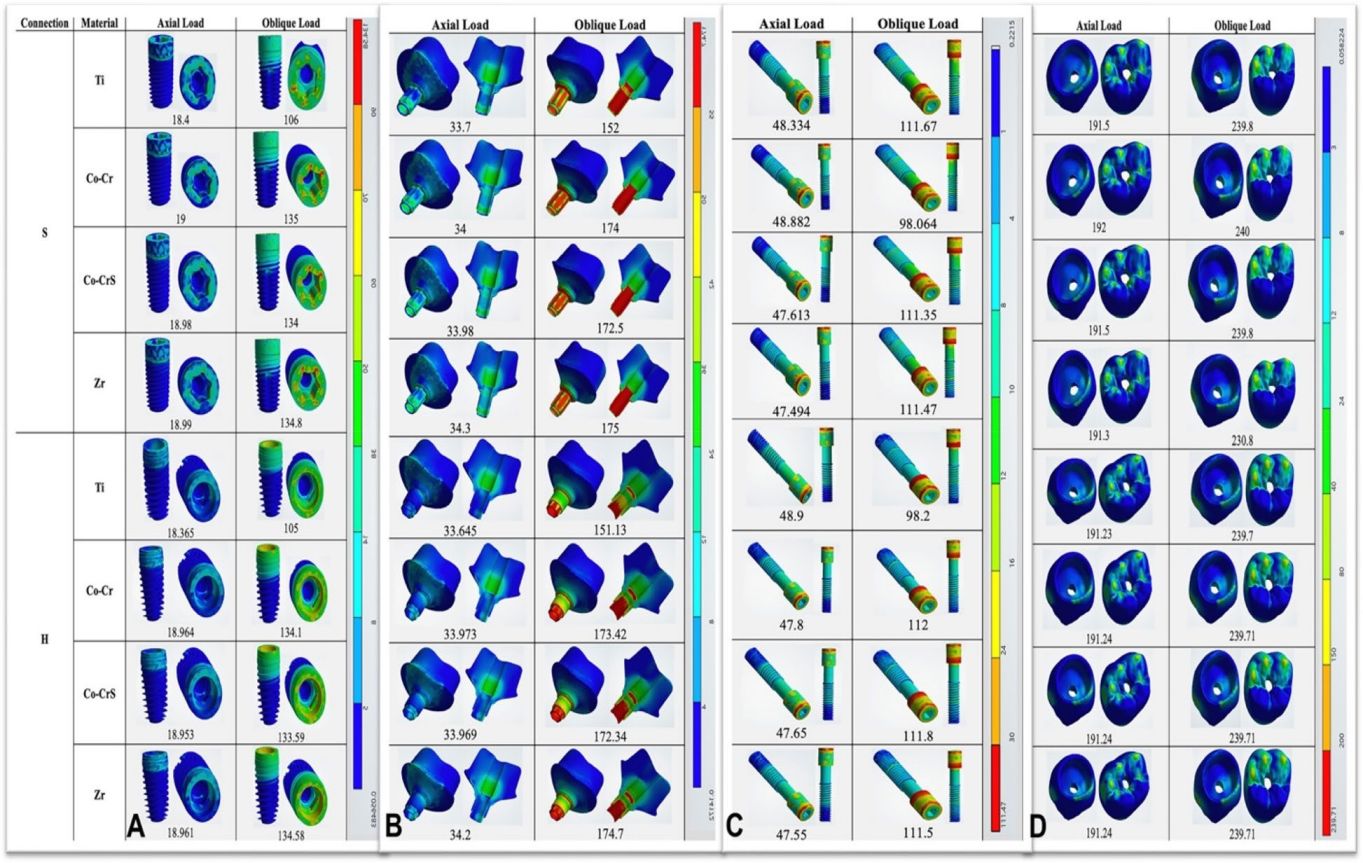
The distribution of the von Mises stress in the implants (**A**), abutments (**B**), screws (**C**), and crowns (**D**) in the models with different connection designs and abutment materials for two loading conditions

**Fig. 8:**
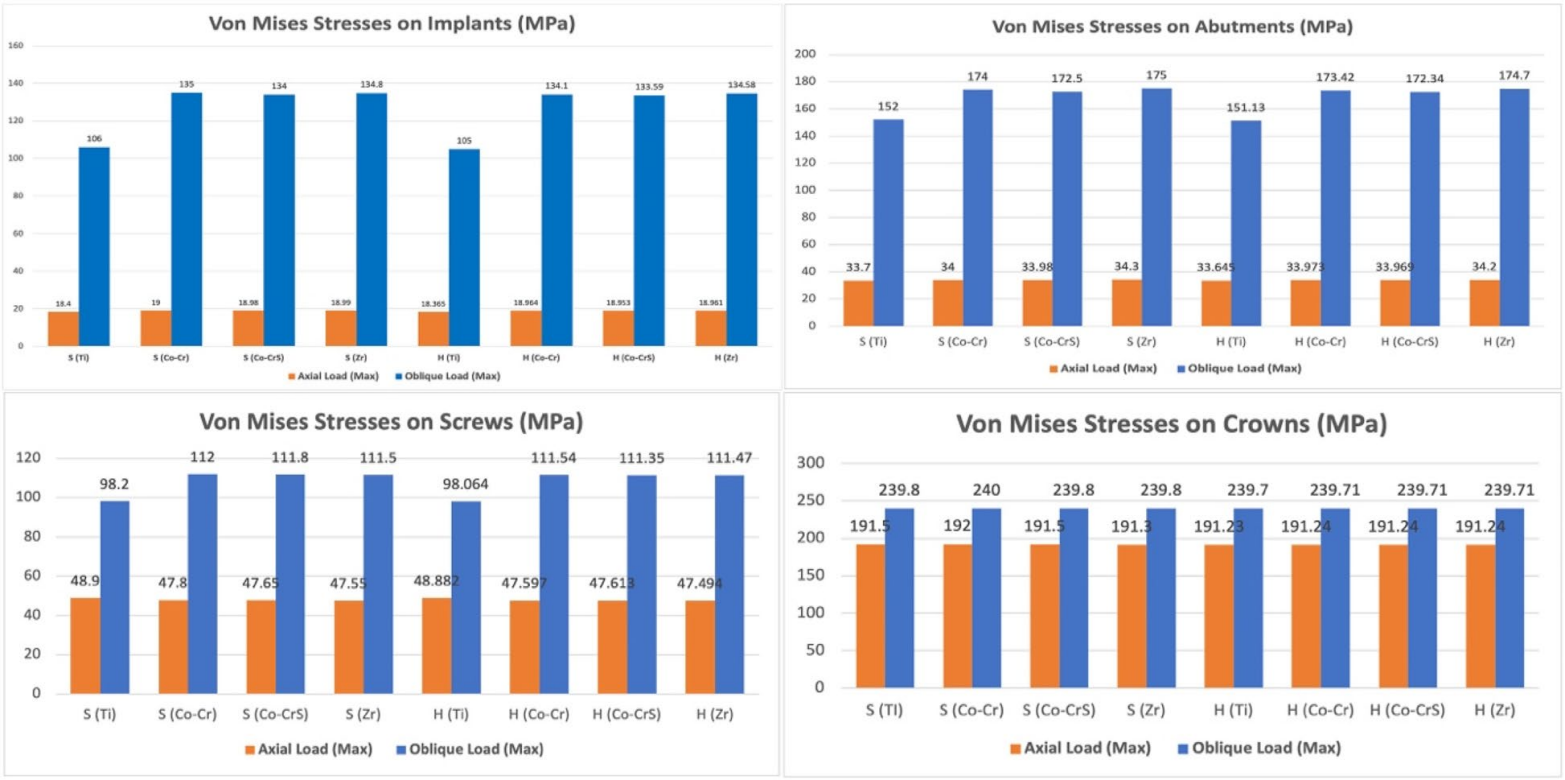
Chart illustrating the variation in von Mises stress (MPa) recorded on the implants, abutments, screws, and crowns of the 16 models under axial and oblique loading appliances

Von Mises stresses were concentrated primarily in the implant neck region across all models (Fig. 7A). Under axial loading, implants demonstrated low stress values, ranging from a minimum of 18.365 MPa in H(Ti) to a maximum of 19.0 MPa in S (Co-Cr). Oblique loading significantly elevated implant stresses, especially in the S (Co-Cr) model, reaching 135.0 MPa. In comparison, titanium abutments in both connections consistently showed lower stress concentrations (106.0 for S and 105.0 MPa for H models) (Figs. 7A and 8).

In both connection types, the highest stresses were located at the abutment neck under axial and oblique loading (Fig. 7B). Stress values were nearly the same for the S and H abutments. Under axial loading, the lowest stress was in the S (Ti) model (33.7 MPa) and the highest in the H (Zr) model (34.2 MPa). Oblique loading caused a marked stress increase, reaching 175.0 MPa in the S (Zr) model. Titanium abutments showed lower stresses under oblique loading, with 152.0 MPa S (Ti) and 151.13 MPa H (Ti) (Fig. 8).

Screw stresses were mainly concentrated in the screw heads and internal threads in both models (Fig. 7C). Under axial loading, stress values were comparable, with the minimum recorded in the H (Zr) model at 47.494 MPa. Oblique loading significantly elevated stresses, attaining 111.8 MPa in the S (Co-Cr) model. Titanium screws showed lower stresses under oblique loading, with 98.2 MPa for S(Ti) and 98.064 MPa for H (Ti) (Figs. 7C and 8).

Both connection types showed comparable stress values for crowns under axial and oblique loading (Figs. 7D and 8). The maximum stress under axial loading was 192.0 MPa in the S (Co-Cr) model. Oblique loading produced higher but similar stresses across all models (~ 240 MPa).

Supplementary figures (S7-S10) present the von Mises stresses of the implant, abutment, and crown, as well as the deformation patterns of the screw, for a comprehensive evaluation.

### Screw deformation

**Fig. 9:**
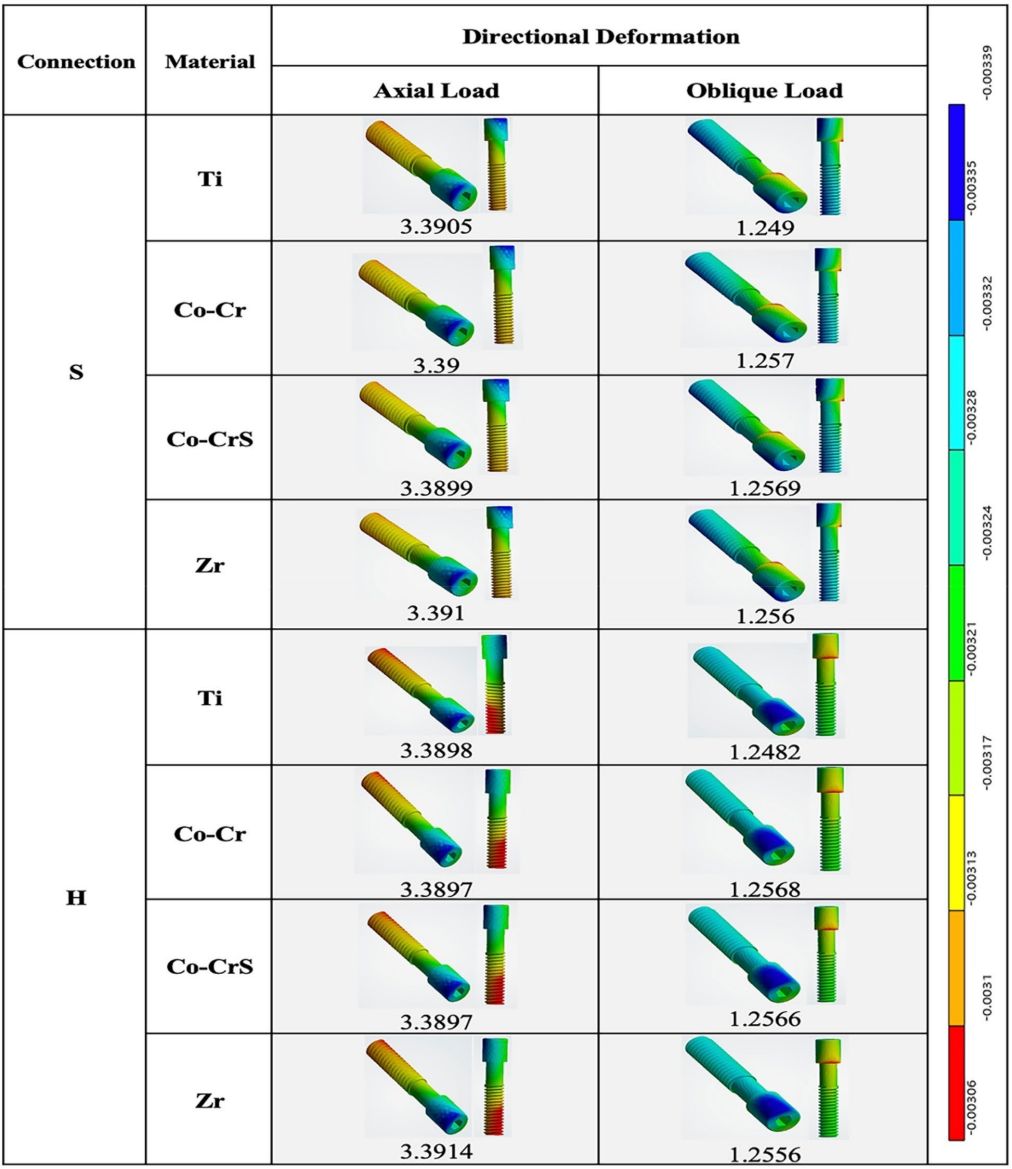
Directional deformation (µm) on the screws in the models with different connection designs and abutment materials for two loading conditions

**Fig. 10:**
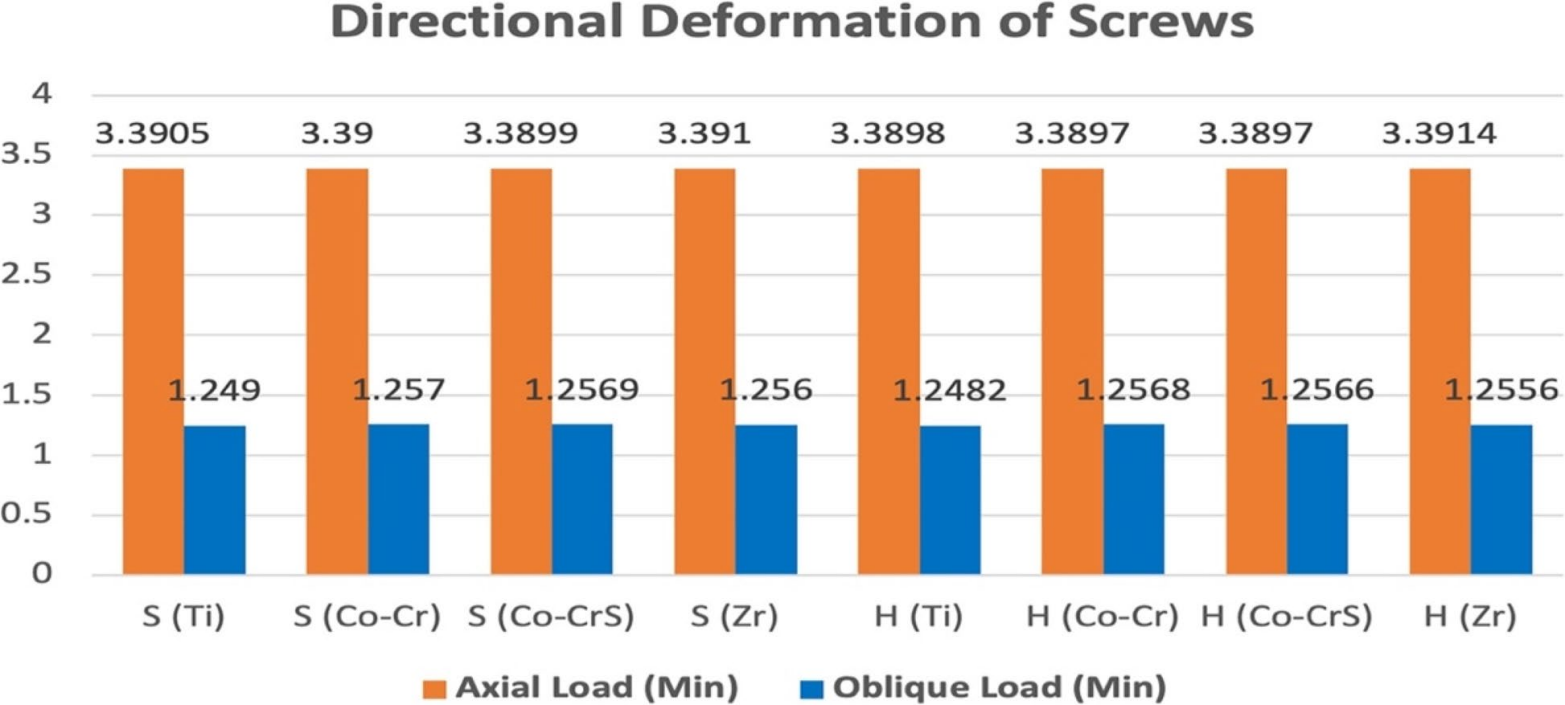
Chart illustrating the variation in directional deformation (µm) recorded on the screw of the 16 models under axial and oblique loadings

Directional screw deformation showed minimal variation across models under axial loading, with the lowest value in H (Co-Cr) and H (Co-CrS) (3.897 µm). Deformation values under oblique loading were consistently lower across all models, with the maximum observed in S (Co-CrS) at 1.257 µm. Stress concentrations were identified at the screw head, base, and thread root, with amplification occurring due to eccentric loading (Fig. 9). No significant differences in screw deformation were observed between models under both loading conditions, indicating comparable mechanical behavior (Figs. 9 and 10).

## Discussion

The FEA provides an understanding of the fundamental mechanics of a specific technical system. It can indicate the internal stress and system vulnerabilities [30]. Dental implant researchers use FEA to assess the stress distribution in critical components and biomechanical performance under various loading circumstances [31, 32]. In the present study, commercial implants were modeled to mimic actual ones. Consequently, this analysis facilitates a more rational selection of a suitable implant connection type and abutment material. However, prior FEA studies often suffer from limitations in model design and validation [33, 34].

In this study, two internal connection designs with differing engineering principles were compared: a star-shaped tube-in-tube design featuring a Torx-like anti-rotational component and cylindrical mating surface, promoting centralized force transmission and consistent seating; and a hybrid design combining a conical interface with an internal hex, valued for its alignment ease and rotational resistance [35, 36]. These systems reflect real-world clinical choices and allow a meaningful analysis of how connection geometry influences the implant biomechanics when paired with different abutment materials.

This study was structured to replicate an optimally managed clinical prosthesis scenario. As the reported average maximum occlusal force in the molar region is 545.7 N, with the maximal masticatory force estimated to be approximately 37–40% [37, 38], the present investigation duplicated maximum occlusal and masticatory forces by delivering a load of 200 N (about 37%). Furthermore, Misch documented standard maximum occlusal forces in the posterior region, varying from 200 to 300 N per implant during regular function [39]. The implant and surrounding bone experience increased stress when force is applied at a singular point [40]. As a result, this investigation employed external stresses at six locations in two distinct orientations: 60 nodes positioned on three buccal cusps and three fossae identified by the precise occlusal position during functional movements to allocate the external loads correctly [41]. While both axial and oblique loads are applied in clinical conditions, oblique loading should be prioritized in analyses, as it represents a realistic occlusal condition and induces significantly higher stress concentrations in cortical bone and implant components than axial loading [42, 43].

The greatest stress concentration transferred to the adjacent bone was noted in the cortical bone. The superior resistance of cortical bone relative to cancellous bone is due to its modulus of elasticity, which is 7–10 times higher, resulting in greater stress concentrations in this area [44]. Gross and Zanatta et al. confirmed that the cortical bone region closest to the cervical area of the implants is the most impacted by occlusal loads, irrespective of bicorticalization [45, 46]. In contrast, softer cancellous bone deforms less under oblique load. As oblique forces do not align with the principal structural axes of cancellous bone (vertical trabeculae), stress is redirected and concentrates in the cortical regions. [47].

The first research hypothesis was partially rejected. The S connection exhibited the lowest axial stress compared to the H connection, with both remaining within the maximum feasible cortical bone stress of 100 MPa [48]. The findings of this study align with previous results, demonstrating that the evaluated implants and connections possess significant capacity for dissipating masticatory loads and exhibit minimal stress concentration in the cortical regions, which could potentially lead to resorption in the marginal bone in the crest area [49, 50]. This might be due to the stable implant-abutment connection permitting stress distribution along the long axis of the implant, thus reducing stress in the cortical bone [32, 51]. Moreover, the cancellous bone of the S implant system exhibited greater maximum stress (average 0.21 MPa) compared to the H implant system (average 0.15 MPa). The H connection might explain this, as it distributes axial forces more evenly and gradually through the conical interface that offers an intimate fit**.**

In contrast, S connection might tend to localize stress at the star corners and threads, transferring higher peak stresses to the cancellous cone, which could not distribute them as efficiently. Markedly decreased stresses were recorded for H models under oblique loading. This decrease could be explained by the H model's conical design, which enhances self-locking and friction under oblique loading, increasing radial engagement and converting lateral forces into axial compression. The result improved implant-abutment stability, reduced micromovement, and led to lower peak stresses compared to axial loading. Nonetheless, both connection types exhibited stresses below the maximal stress threshold (5.0 MPa) for the onset of cancellous bone resorption [52].

During axial loading, both S and H implant-abutment connections produced strain levels within physiological safety limits: 50–1500 με for cancellous bone and 200–2500 με for cortical bone [53, 54]. In contrast, oblique loading resulted in strain values that exceeded safe thresholds for connection types and all abutment materials. Cortical bone showed high strain levels above 6000 με that would induce bone remodeling as mechanical strain reached a threshold sufficient to stimulate cellular response, reflecting stress transmission through bone tissue [54, 55]. In contrast, cancellous bone exhibited reduced strains under identical conditions. The observed discrepancy can be attributed to the increased stiffness of the cortical bone, resulting in greater absorption of oblique forces.

The present investigation revealed that both connections exhibited nearly similar stresses on the implant, abutment, crown, and screw that did not exceed the yield strength of titanium alloy (780–950 MPa) [56]. Therefore, the second hypothesis was rejected. The star-shaped tube-in-tube implant-abutment connection represents a biomechanical innovation to enhance rotational stability and optimize stress distribution throughout the implant assembly (https://www.bredent-implants.com/wp-content/uploads/2023/12/Prospekt-SKY-Implantatsystem_009991GB_20231206_low-.pdf). The multi-lobed internal design enhances the contact surface area between the implant and abutment, optimizes load distribution across several lobes rather than just the screw shank, redirects load paths from the screw threads to the implant body, and reduces micromovement under occlusal loads. A star design may facilitate a more concentrated axial load transmission, reducing interface shear stresses. This led to less compressive and tensile stress in the peri-implant bone along the cortical boundary, relative to H groups, facilitating crestal bone preservation and possibly mitigating the risk of marginal bone loss. This is based on a previous study that reported that the tube-in-tube connection type exhibited lower stress values in most loading and tilting simulations in the implant components and the peripheral bone [57].

The hybrid Morse taper with internal hex implant-abutment connection tends to transfer more load to the surrounding cortical bone than the star-shaped tube-in-tube connection, possibly due to the lower compressive strength of the internal hex within a Morse taper configuration. Studies have shown that Morse taper systems incorporating an internal hexagon can exhibit significant interface distortion [58]. However, some findings indicated that a conical connection with an internal hex results in minimal stress on both the implant and surrounding bone [59]. The Morse taper system operates via surface friction, known as cold welding, where microscopic surface roughness induces pressure that causes fusion of the surfaces, creating a near-integral connection. This promotes uniform stress distribution across the implant-abutment connection [30]. The findings align with the study by Lin et al. and Hansson et al., who observed that conical implant-abutment connection systems exhibited superior performance as a force transmission system compared to internal hexagonal and external flat-top systems [60, 61]. However, other studies indicated increased von Mises stresses in the neck region of the abutment-prosthesis complex, alongside a notable abutment fracture rate of 2.2%, with all fractures localized to the abutment neck and screw [62, 63].

The deformation of the prosthetic abutment screw may lead to mechanical instability and elevate the risk of screw loosening over time. The current study found that both connection groups displayed comparable directional deformation patterns when subjected to axial and oblique loading conditions. The overall magnitude of deformation was minimal, likely attributable to the implant's inherent rigidity, abutment, crown materials, and precise fit of the implant components. The aforementioned factors collectively improve the mechanical stability of the prosthetic assembly. This finding aligns with earlier research indicating that screw loosening is less prevalent in Morse taper with internal hex connections than in octagon designs, due to the decreased separation between the screw and the internal abutment surface when subjected to load [64]. Therefore, it was observed that less stress was transferred to the screw. However, it was reported that masticatory forces up to 550 N were well within the abutment screw's capacity to sustain extended service life and maintain its elastic behavior [65]. However, abutment screw deformation is influenced by several factors, including screw geometry, design variables, material properties, applied torque, loading condition, implant diameter, and the presence of a micro gap that may potentially lead to screw loosening or fracture [33, 66].

The third hypothesis was rejected. The Ti abutment exhibited the most favorable performance in both connection groups under oblique loading. Titanium is distinguished by remarkable ductility, enabling titanium abutments to absorb and redistribute loads, thus mitigating catastrophic failure. Titanium might bend or deform under excessive stress instead of fracturing. The elastic modulus of titanium (110 GPa) is more akin to that of bone (13–20 GPa) [67]. This higher stiffness alignment enhances stress distribution, consequently diminishing stress accumulation at the bone-implant interface, which reduces the risk of bone resorption and demonstrates greater stress dispersion and fatigue resistance [14]. Biomechanical advantages are substantiated by randomized controlled trials and prospective studies demonstrating high survival rates and soft tissue stability associated with titanium CAD/CAM abutments [68, 69].

However, Co-Cr and soft milled models demonstrated moderate stress values that exceeded Ti's. A multicenter retrospective study of Co-Cr single crowns indicated a cumulative survival rate of approximately 90.3% at 5 years, with minimal material-related complications [70]. Comprehensive data on ceramic-veneered Co-Cr fixed prostheses demonstrate a 5-year survival rate of 92.8%, highlighting their clinical effectiveness [71]. The clinical observations correspond with contemporary biomechanical findings demonstrating advantageous soft-milled Co-Cr–Mo stress distribution patterns. Co-Cr demonstrates a higher elastic modulus and greater strength than titanium, albeit with reduced ductility. This characteristic may enhance stress transfer to the adjacent bone, potentially increasing the risk of stress shielding and remodeling [72]. Additionally, the reduced biocompatibility of Co-Cr alloys relative to titanium and zirconia raises concerns, particularly in individuals with metal hypersensitivity, since it may elicit allergic reactions or peri-implant inflammation [16].

Zirconia abutments demonstrate stress levels similar to those of Co-Cr abutments, affirming their safety and aesthetic benefits. A 13-year randomized controlled trial comparing zirconia and titanium abutments demonstrated 100% survival rates for both implants and restorations, with no significant differences observed in biological or esthetic outcomes [73]. Systematic reviews indicate a high implant survival rate of approximately 96%, accompanied by marginal bone loss and probing depths comparable to, or in some instances slightly superior to, those associated with titanium [74]. While zirconia may provide superior esthetic outcomes in patients with thin gingival biotypes, differences in survival and biological performance are minimal [75, 76]. However, zirconia’s ability to undergo plastic deformation is limited; it can fracture suddenly and unpredictably if its tensile strength is exceeded. FEA frequently reveals elevated tensile stresses in critical areas, specifically at the cervical margin of the abutment under oblique loading and at the abutment-implant connection [77]. However, zirconia demonstrates a significantly greater elastic modulus than bone, with values of 200 GPa for zirconia and 13 GPa for cortical bone. This disparity may result in stress shielding, where the diminished load is transferred to the bone, potentially leading to bone resorption and localized stress transfer, which increases the risk of mechanical overload [78, 79].

Although stress distribution varies depending on abutment material, clinical outcomes indicate that titanium, Co-Cr, and zirconia abutments can all be successfully used, with the choice guided by functional demands, esthetic goals, and cost considerations.

### Limitations

Although this investigation is founded upon an FEA that simulates mechanical behavior, it relies on idealized assumptions. The model did not account for the dynamics of soft tissue, the process of bone remodeling, or the individual occlusal forces. ial properties were assumed to be linearly elastic, homogeneous, and isotropic, which may not accurately represent in vivo conditions. Furthermore, this study neglects to address long-term fatigue behavior and does not account for possible manufacturing flaws in customized abutments. The loading conditions were static and applied at specific locations, whereas real-world masticatory loads are more dynamic and multi-directional.

Additionally, stress distribution is influenced by parameters that extend beyond the geometry of the implant and abutment, such as the number of components (one- versus two-piece), screw dimensions, thread design, material, and contact area, which were not considered in the current study [80]. FEA data alone is insufficient for predicting clinical outcomes, such as aesthetics, long-term survival, and soft tissue health. Future research should integrate clinical follow-ups and fatigue testing to validate these biomechanical findings.

## Conclusions

The star-shaped tube-in-tube and hybrid Morse taper with internal hex connections exhibited comparable stress distribution across implant components, with stresses remaining below the yield strength of titanium alloy, signifying a minimal risk of mechanical failure. Both connections induced safe stress levels in the surrounding bone but caused cortical bone strains exceeding the safe limit under oblique loading. Zirconia, Co-Cr, and soft-milled Co-Cr abutments exhibited moderate, evenly distributed stresses, while titanium abutments provided the most favorable stress distribution. Both connection types also showed minimal screw deformation, suggesting reduced risk of screw loosening.

## Supplementary Information

Supplementary Material 1.

## Data Availability

The datasets used and/or analysed during the current study are available from the corresponding author on reasonable request.
