# Supplementary material for: Influence of different customized abutment materials on stress distribution of different internal implant-abutment connections: finite element analysis
Source: BMC Oral Health. 2025 Nov 22;25:1820. doi: 10.1186/s12903-025-07122-8 (PMC12640568; doi:10.1186/s12903-025-07122-8)
Supplement: Supplementary file 1 — Supplementary Material 1. [file 12903_2025_7122_MOESM1_ESM.docx]

**Supplementry Figures**

**Fig. S1** The distribution of the maximum principal stresses on the cortical bone in the models with different connection designs and abutment materials for two loading conditions.

**Fig. S2** The distribution of the minimum principal stresses on the cortical bone in the models with different connection designs and abutment materials for two loading conditions.

**Fig. S3** The distribution of the maximum principal stresses on the cancellous bone in the models with different connection designs and abutment materials for two loading conditions.

**Fig. S4** The distribution of the minimum principal stresses on the cancellous bone in the models with different connection designs and abutment materials for two loading conditions.

**Fig. S5** The distribution of the maximum principal strains on the cancellous bone in the models with different connection designs and abutment materials for two loading conditions.

**Fig. S6** The distribution of the minimum principal strains on the cancellous bone in the models with different connection designs and abutment materials for two loading conditions.

**Fig. S7** The distribution of the von Mises stress in the implants in the models with different connection designs and abutment materials for two loading conditions.

**Fig. S8** The distribution of the von Mises stress in the abutments and implant-abutment connection in the models with different connection designs and abutment materials for two loading conditions.

**Fig. S9** The distribution of the von Mises stress in the screws in the models with different connection designs and and abutment materials for two loading conditions.

**Fig. S10** The distribution of the von Mises stress (Mpa) in the crowns in the models with different connection designs and and abutment materials for two loading conditions.
